# A review on laser-assisted veneer debonding

**DOI:** 10.1007/s10103-026-05052-7

**Published:** 2026-10-05

**Authors:** Ioana Duncea, Cristina Muresan, Oana Almasan, Smaranda Buduru, Cecilia Bacali

**Affiliations:** Department of Prosthodontics, Iuliu Hațieganu University of Medicine and Pharmacy, Cluj-Napoca, Romania

**Keywords:** Laser debonding, Er:YAG laser, Er,Cr:YSGG laser, Veneer debonding, Debonding time, Debonding efficiency, laser-tissue interaction

## Abstract

This literature review examines the advancements in laser technology that have enhanced the veneer debonding process, offering insights into the proper implementation of these techniques in clinical practice with emphasis on laser parameters, debonding efficiency, thermal effects, and preservation of tooth structure. A systematic literature search was conducted on PubMed,Web of Science, Scopus and Cochrane Library. The review was structured according to the PICO framework. The search was performed independently by two reviewers on laser-assisted debonding of ceramic veneers using Er:YAG or Er,Cr:YSGG lasers.Based on a comprehensive evaluation of existing studies, the review presents different types of prosthetic restorations, materials, and laser parameters, and how they interact with each other. The benefits of laser precision, including improved control, workflow efficiency, fewer procedural steps, and reduced tissue impact, are emphasised to help dental practitioners enhance the standard of care in veneer debonding. Across the twenty nine studies that were obtained, laser debonding facilitated removal while pulpal temperature increases remained below commonly cited critical values. Both Er:YAG and Er,Cr:YSGG systems demonstrated effective veneer removal. Thinner veneers nedeed less irradiation time and lower energy. Limited enamel alterations occured when appropriate parameters were used. Current studies suggest that Er:YAG and Er, Cr: YSGG lasers offer clinical advantages in terms of efficiency, safety, and patient comfort. Studies demonstrate that optimized laser settings reduce debonding time and minimize the risk of thermal damage to underlying tooth structure and surrounding tissues. Debonding efficiency was influenced by veneer thickness, ceramic type, cement composition, laser power, pulse duration and cooling protocols Er:YAG and Er,Cr:YSGG lasers are promising tools for ceramic veneer debonding. However, results obtained in these studies cannot be directly associated to patient-centered issuses, further standardized clinical studies are required before definitive clinical recommendations can be made.

## Introduction

Modern dentistry has advanced significantly in terms of procedure accuracy, patient comfort, and overall clinical effectiveness with the introduction of laser technology [1, 2]. The use of lasers enables a more predictable and patient-friendly approach, enhancing the overall quality of dental care.

Veneer debonding, a critical but delicate procedure in restorative dentistry, has historically been performed by mechanical means using burs a specialized instruments [3]. Despite their effectiveness, traditional methods have well-documented disadvantages, including risk of damage to underlying tooth tissues, patient discomfort, and variable outcomes [3].

Recent developments in laser technology present a viable alternative to these conventional techniques [4]. Lasers provide a more sophisticated method of veneer removal by directing targeted energy with remarkable precision [5]. This shift aligns with the trend in dentistry toward less invasive, more patient-centered care [4]. In particular, Eerbium-doped lasers have been shown to improve debonding efficiency by reducing procedure time while minimizing heat transfer to adjacent tissues, thereby preserving tooth structure [6]. Their selectivity in interacting with dental materials facilitates faster and easier veneer removal, with the potential for less structural damage, reduced patient pain, and better overall procedural outcomes [6].

Despite these advances, several challenges remain. The interaction between laser energy and tooth tissues is governed by multiple biophysical parameters, laser type, energy settings, and veneer material properties, all of which must be understood and carefully managed to optimize outcomes while ensuring patient safety [7]. The initial cost of laser systems and the need for specialized training present additional barriers to clinical adoption [1].

This literature review provides a comprehensive analysis of recent studies on laser-assisted veneer debonding. Specifically, it aims to: evaluate the effectiveness of laser-assisted debonding techniques compared to traditional mechanical methods; identify optimal laser parameters for safe and efficient veneer removal; examine laser-tissue interaction mechanisms relevant to clinical practice; highlight practical considerations and future directions for research.

The term LASER is an acronym for "Light Amplification by Stimulated Emission of Radiation." The conversion of laser energy into heat causes precise alterations in target tissue — a process known as laser-tissue interaction [8]. This interaction is influenced by the laser's physical characteristics and the biological properties of the target tissue.

Erbium-doped lasers (Er:YAG), CO2 lasers, and Nd:YAG lasers can degrade resinous bonding materials through three mechanisms:

Erbium-doped lasers (Er), CO2 lasers, and Nd lasers can degrade resinous bonding materials through thermal softening, thermal ablation, and photoablation. Thermal softening occurs when laser energy heats the resin, resulting in material softening. Thermal ablation involves the rapid vaporization of the resinous adhesive. Photoablation occurs through the interaction of laser energy with the resin, elevating atomic energy levels to the point of bond dissociation and material decomposition.

The Er:YAG laser, which relies primarily on thermal ablation and photoablation, is preferred over CO2 and Nd:YAG lasers, which tend toward thermal softening and carry a greater risk of collateral damage [9]. In a documented clinical case conducted by Dr. Calabro, the patient did not require anaesthesia and reported no sensitivity during or after the debonding procedure [10].

Regardless of the technique employed, veneer debonding presents several inherent challenges. One of the principal concerns is the risk of damage to the underlying tooth structure, including accidental enamel damage and increased tooth sensitivity, particularly in cases where enamel integrity was already compromised before veneer placement [10]. Adhesive removal also remains technically demanding, as modern resin cements are formulated for long-term durability and may require considerable time for complete elimination [11]. Veneer preservation represents another important challenge, especially because thinner veneers are vulnerable to fracture throughout the treatment cycle, and fracture during removal precludes reuse [12]. Patient discomfort must also be considered, since the noise, vibration, and pressure associated with debonding can cause anxiety, even in the absence of pain [13]. Finally, laser-assisted debonding requires specific equipment and a high level of clinical skill to avoid iatrogenic damage [13].

From a biomechanical perspective, the interplay between tissue sensitivity, material properties, and mechanical forces adds further complexity [14]. Residual adhesive remaining after debonding can compromise subsequent restoration [15]. Strategies to mitigate these challenges include optimizing adhesive protocols, incorporating stress-relieving techniques, and leveraging advanced imaging modalities to assess structural integrity before and after debonding [14].

## Methods

### Information sources and search strategy

An extensive literature search was conducted on four major databases widely used for biomedical and dental research (PubMed, Web of Science, Scopus and Cochrane Library). The search was performed independently by two reviewers. The strategy was designed to find the relevant studies within the scope of this review.

The following Boolean search string was applied across databases: *("veneer" OR "dental veneer" OR "porcelain veneer") AND ("debonding" OR "removal" OR "detachment" OR "separation") AND ("laser" OR "laser-assisted" OR "laser technology" OR "laser application")* (Table 1).

**Table 1:** Database search strategy and results

Database/Source	Search Terms/Strategy	Results
PubMed	((veneer) OR (dental veneer) OR (porcelain veneers)) AND ((debonding) OR (removal)) AND ((laser) OR (laser-assisted))	41
The Cochrane Library	((veneer) OR (dental veneer) OR (porcelain veneers)) AND ((debonding) OR (removal)) AND ((laser) OR (laser-assisted))	3
SCOPUS	(TITLE-ABS-KEY ("veneer" OR "dental veneer" OR "porcelain veneer") AND TITLE-ABS-KEY ("debonding" OR "removal" OR "detachment" OR "separation") AND TITLE-ABS-KEY ("laser" OR "laser-assisted" OR "laser technology" OR "laser application"))	62
WEB OF SCIENCE	veneer OR dental veneer OR porcelain veneer (All Fields) and debonding OR removal OR detachment OR separation (All Fields) and laser OR laser-assisted OR laser technology OR laser application (All Fields)	59
*Total*	—	*165*

The review was structured according to the PICO framework (Table 2) The population included patients receiving dental veneer treatment, as well as studies conducted on extracted human or bovine teeth. The intervention of interest was laser-assisted debonding using Er,Cr, or Er lasers, while the comparison was conventional mechanical debonding. The primary outcomes were debonding time, bond strength after debonding, and pulpal temperature. Secondary outcomes included enamel damage, post-operative sensitivity, and other complications.

**Table 2:** PICO Framework

Component	Description
Population	Human teeth and bovine teeth restored with ceramic veneers (in vitro/ex vivo models)
Intervention	Veneer debonding using laser systems
Comparison	Different laser parameters, veneer thicknesses, ceramic materials, resin cements Mechanical debonding
Outcomes	Debonding time, bond strength reduction, pulpal temperature changes, enamel damage, restoration integrity

### Eligibility criteria

Inclusion criteria: english language, original research articles or clinical trials reporting quantitative or qualitative data on laser-assisted veneer debonding using lasers; full-text availability.

Exclusion criteria: review articles (to prevent duplication), studies without full-text access, and studies not reporting outcomes relevant to the research questions.

Study selection followed PRISMA guidelines. From 165 identified records, 74 duplicate were removed. After screening 91 records, 39 were excluded, leaving 52 for full-text review. Of these, 3 were not retrieved and reamaning 49 reports assessed for eligibility. 20 were excluded as mentined in Prisma table, resulting 29 studies included in the qualitative synthesis. The selection of relevant studies is highly important for the final evidence base. Studies frequently omitted critical safety metrics like intrapulpal temperature changes or quantitative bond strength reduction, leading to lack of standardized reports in laser debonding research. Standardizing reporting guidelines for in vitro dental research is essential to ensure that future studies consistently report key parameters, which will improve data synthesis and provide more robust evidence to support clinical practice.

**Fig. 1:**
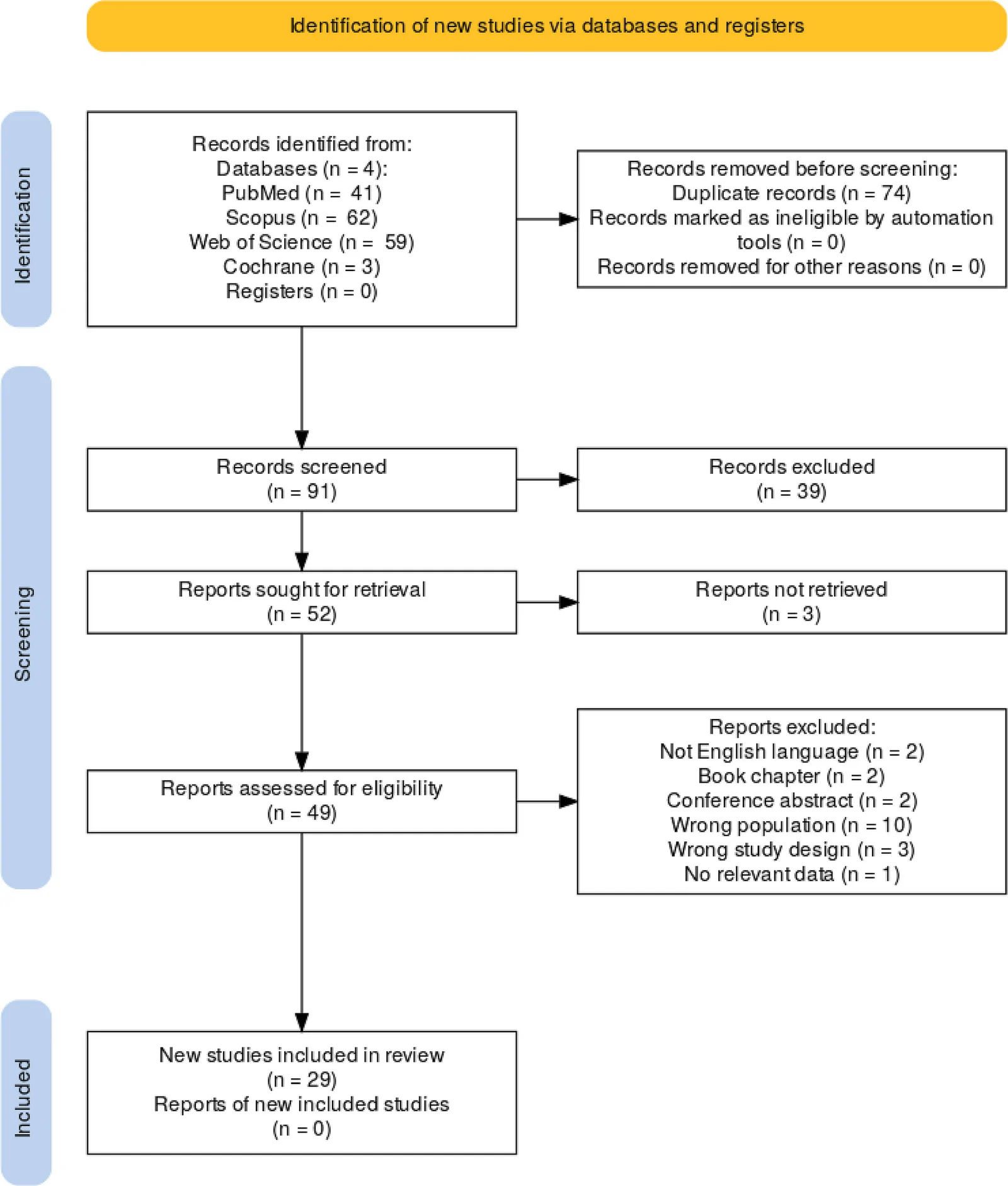
PRISMA flow summary

The selection process is summarized in Fig. 1.

### Study selection

Two independent reviewers conducted data extraction. The following data were systematically collected: study author(s) and year; study design; veneer material type (e.g., lithium disilicate, feldspathic); laser parameters (type, power, pulse duration, exposure time, cooling method); and outcome measures (debonding time, bond strength, pulpal temperature, enamel damage, post-operative sensitivity, complications).

Due to heterogeneity across included studies a qualitative synthesis was performed. Studies were grouped by laser type (Er,Cr:YSGG vs. Er:YAG) and restoration type, then compared across key outcomes: debonding time, bond strength, and thermal impact on the dental pulp.

### Risk of bias (quality assessment)

Several measures were applied to mitigate potential bias. Selection bias was addressed through the systematic application of predefined inclusion and exclusion criteria. Data representation bias was reduced by including studies involving a range of laser types, power settings, and veneer materials. Database bias was minimized through multi-database searching. Parameter bias was addressed by prioritizing studies that provided detailed reporting of laser settings.

Twenty nine included studies were in vitro or ex vivo laboratory and clinical studies.

The QUIN Tools were applied as the best available validated tools for in vitro or ex vivo studies, consistent with their use in published systematic reviews of dental in vitro research. The assessment comprised 12 methodological criteria, with each item recorded as a numerical score (0–2) according to the completed study-level evaluations. Individual item scores were summarized as a total score and percentage, together with the corresponding quality category for each article (Table 3, Table 3.1, Table 3.2, Table 3.3, Table 3.4, Table 3.5).

**Table 3:** QUIN assessment of included in vitro studies

Table 3.1. QUIN risk of bias assessment for in vitro studies								
No	Criteria	AlBalkhi & Hamadah (2022) [16]	Oztoprak et al. (2012) [17]		Al-Araji & Sulaiman (2022) [18]	Buu et al. (2010) [19]	Walinski et al. (2021) [20]	Al-Karadaghi & Jawad (2023) [21]
1	Clearly stated aims/objectives	2	2		2	2	2	2
2	Detailed explanation of sample size calculation	0	0		0	0	0	0
3	Detailed explanation of sampling technique	1	1		1	1	1	1
4	Details of comparison group	1	1		1	0	0	1
5	Detailed explanation of methodology	1	1		1	1	1	1
6	Operator details	2	2		2	2	2	2
7	Randomization	2	2		2	2	2	2
8	Method of measurement of outcome	2	2		2	2	2	2
9	Outcome assessor details	0	0		0	0	0	0
10	Blinding	2	2		2	0	2	2
11	Statistical analysis	2	2		2	2	2	2
12	Presentation of results	2	0		2	0	2	2
Total score		17/24	15/24		17/24	12/24	16/24	17/24
Risk of bias score (%)		70.83%	62.5%		70.83%	50.00%	66.67%	75.00%
Quality category		Low Risk of Bias (High Quality)	Low Risk of Bias (High Quality)		Low Risk of Bias (High Quality)	Medium risc of bias high quality	Medium risk of bias	Low Risk of Bias (High Quality)
Table 3.2 QUIN risk of bias assessment for in vitro studies								
.No		Criteria		Eid et al. (2021) [22]	Giraldo-Cifuentes et al. (2020) [23]	Alikhasi et al. (2019) [24]	El-Damanhoury et al. (2022) [25]	Iseri et al. (2014) [26]
1		Clearly stated aims/objectives		2	2	2	2	2
2		Detailed explanation of sample size calculation		0	0	0	0	0
3		Detailed explanation of sampling technique		1	1	1	1	1
4		Details of comparison group		0	1	0	0	1
5		Detailed explanation of methodology		1	1	1	1	1
6		Operator details		2	2	2	2	2
7		Randomization		2	2	2	2	2
8		Method of measurement of outcome		2	2	2	2	2
9		Outcome assessor details		0	0	0	0	0
10		Blinding		2	2	2	2	2
11		Statistical analysis		2	2	2	2	2
12		Presentation of results		2	2	2	2	2
Total score				16/24	17/24	16/24	16/24	17/24
Risk of bias score (%)				66.67%	70.83%	66.67%	66.67%	70.83%
Quality category				Medium Risk of Bias	Low Risk of Bias (High Quality)	Medium Risk of Bias	Medium Risk of Bias	Low Risk of Bias (High Quality)
Table 3.3 QUIN risk of bias assessment for in vitro studies								
No		Criteria		Yilmaz et al. (2019) [27]	AlBalkhi, Swed & Hamadah (2018) [28]	Amin (2023) [29]	Zhang et al. (2018) [30]	Giraldo Cifuentes et al. (2020) [31]
1		Clearly stated aims/objectives		2	2	2	2	2
2		Detailed explanation of sample size calculation		0	0	0	0	0
3		Detailed explanation of sampling technique		1	1	2	1	1
4		Details of comparison group		1	1	1	0	0
5		Detailed explanation of methodology		1	1	1	1	1
6		Operator details		2	2	2	2	2
7		Randomization		2	2	2	2	2
8		Method of measurement of outcome		2	2	2	2	2
9		Outcome assessor details		0	0	1	0	0
10		Blinding		2	2	2	1	2
11		Statistical analysis		2	2	2	2	2
12		Presentation of results		2	2	2	2	2
Total score				17/24	17/24	19/24	15/24	18/24
Risk of bias score (%)				70,83%	70,83%	79.17%	62.50%	75.00%
Quality category				Low Risk of Bias (High Quality)	Low Risk of Bias (High Quality)	Low Risk of Bias (High Quality)	Medium Risk of Bias	Low risk of bias, high quality
Table 3.4. QUIN risk of bias assessment for in vitro studies								
No		Criteria		El-Sheikh et al. (2024) [32]	Al-Karadaghi et al. (2023) [33]	Raafat et al. (2025) [34]	Zanini et al. (2020) [35]	Yildiz et al. (2023) [36]
1		Clearly stated aims/objectives		2	2	2	2	2
2		Detailed explanation of sample size calculation		2	0	2	0	2
3		Detailed explanation of sampling technique		2	1	2	1	1
4		Details of comparison group		1	1	1	1	1
5		Detailed explanation of methodology		1	1	1	2	1
6		Operator details		2	2	2	2	2
7		Randomization		2	2	2	2	2
8		Method of measurement of outcome		2	2	2	2	2
9		Outcome assessor details		0	0	0	1	0
10		Blinding		2	2	2	2	2
11		Statistical analysis		2	2	2	2	2
12		Presentation of results		2	2	2	2	2
Total score				20/24	17/24	20/24	19/24	19/24
Risk of bias score (%)				83.33%	70.83%	83.33%	79.17%	79.17%
Quality category				Low risk of Bias high quality	Low risk of bias high quality	Low risk of Bias, High Quality	Low risk of Bias high quality	Low risk of bias, high quality
Table 3.5 QUIN risk of bias assessment for in vitro studies								
No		Criteria		Abdulwahhab et al. (2025) [37]	Culhaoglu et al. (2021) [38]	Xiong et al. (2025) [39]	Tak et al. (2015) [40]	Hussein et al. (2021) [41]
1		Clearly stated aims/objectives		2	2	2	2	2
2		Detailed explanation of sample size calculation		0	0	0	0	0
3		Detailed explanation of sampling technique		1	1	1	0	1
4		Details of comparison group		1	1	1	0	1
5		Detailed explanation of methodology		2	1	1	2	1
6		Operator details		2	2	2	2	2
7		Randomization		2	2	2	2	2
8		Method of measurement of outcome		2	2	2	2	2
9		Outcome assessor details		0	0	0	0	0
10		Blinding		2	2	0	2	2
11		Statistical analysis		2	2	2	2	2
12		Presentation of results		2	2	2	2	2
Total score				18/24	17/24	15/24	16/24	17/24
Risk of bias score (%)				75.00%	70.83%	62.5%	66.67%	70.83%
Quality category				Low risk of Bias,high quality	Low risk of bias, high quality	Medium risk of bias	Medium risk of bias	Low risk of bias, high quality

The three clinical publications were critically appraised using the JBI Critical Appraisal Checklist for Case Series (Table 4). This tool was selected because the included clinical evidence is case-based research: van As (2012) [42] presents three clinical cases, Kursoglu and Gursoy (2013) [43] report two clinical cases, and Deeb, Grzech-Lesniak and Bencharit (2023) [44] describe a retrospective clinical case series.

**Table 4:** JBI Case series assessment of included clinical studies

Clinical study designs				
Study	Design		Appraisal tool	
Van As (2012) [42]	Clinical case-based report; 3 cases		JBI Case Series	
Kursoglu & Gursoy (2013) [43]	Report of 2 clinical cases		JBI Case Series	
Deeb, Grzech-Lesniak & Bencharit (2023) [44]	Retrospective clinical case series; 29 patients		JBI Case Series	
No	JBI criterion	Van As (2012) [42]	Kursoglu & Gursoy (2013) [43]	Deeb et al. (2023) [44]
1	Were there clear criteria for inclusion in the case series?	No	No	Unclear
2	Was the condition measured in a standard, reliable way for all participants included in the case series?	Unclear	Yes	Yes
3	Were valid methods used for identification of the condition for all participants included in the case series?	Yes	Yes	Yes
4	Did the case series have consecutive inclusion of participants?	No	Unclear	Unclear
5	Did the case series have complete inclusion of participants?	No	Unclear	Unclear
6	Was there clear reporting of the demographics of the participants in the study?	Yes	Yes	Yes
7	Was there clear reporting of clinical information of the participants?	Yes	Yes	Yes
8	Were the outcomes or follow-up results of cases clearly reported?	Yes	Yes	Yes
9	Was there clear reporting of the presenting site(s)/clinic(s) demographic information?	Unclear	Yes	Yes
10	Was statistical analysis appropriate?	N/A	N/A	Yes
	Overall appraisal	Include with caution	Include with caution	Include with caution

Interpretation

- Van As (2012) [42]: Detailed clinical descriptions, intervention parameters and outcomes are provided, but the three cases were selected as illustrative cases and no formal eligibility, consecutive inclusion or complete inclusion process was reported.
- Kursoglu & Gursoy (2013) [43] The two patients, clinical history, veneer fracture, laser protocol and follow-up are clearly described. However, eligibility criteria and whether the cases were consecutive or represented all eligible cases were not reported.
- Deeb et al. (2023) [44]: The paper explicitly describes a retrospective clinical case series with a standardized laser-assisted retrieval protocol and detailed demographic, clinical and outcome reporting. The main uncertainty concerns the absence of explicit eligibility criteria and clear reporting of consecutive/complete case inclusion.

## Results

### Search and selection

Twenty nine studies met the inclusion criteria and were included in the qualitative synthesis. Table 5 provides a detailed summary of each study, including restoration type, cement, sample characteristics, laser type, study design, and key findings.

**Table 5:** Summary of included studies on laser-assisted veneer debonding

No	Study	Restoration Type	Cement	Teeth	Laser	Study Details	Key Findings
1	AlBalkhi & Hamadah (2022) [16]	Porcelain laminate veneers	Variolink N (Ivoclar Vivadent)	36 maxillary premolars in 6 groups	Er:YAG (2940 nm)	Investigated influence of pulse duration and water/air cooling ratio on laser efficiency	Optimal energy density settings resulted in efficient veneer removal with minimal heat generation. Er:YAG laser reduced bond strength. Cooling ratio critical for safety
2	Oztoprak et al. (2012) [17]	Empress II ceramic laminate veneer specimens, 0.7 mm thick × 5 mm diameter	Variolink II (Ivoclar Vivadent)	80 extracted bovine mandibular incisors	Er:YAG	In vitro study. Four groups (*n =* 20): control and laser scanning for 3, 6, or 9 s. One second after irradiation, mechanical force was applied and shear bond strength was measured	All three laser application times significantly reduced debonding strength versus control. Er:YAG scanning was effective for veneer debonding by softening the adhesive resin
3	Al-Araji & Sulaiman (2022) [18]	Lithium disilicate (IPS e.max Press) veneer discs, 0.5 mm and 1 mm thick	Light-cured resin cement (Variolink Esthetic LC, Ivoclar Vivadent) with Adhese Universal adhesive	40 extracted bovine teeth	Er,Cr:YSGG	In vitro study. Four groups (control/laser × two thicknesses); laser applied at 2.5 W, 25 Hz for 60 s using scanning technique; shear bond strength and ARI failure mode evaluated	Laser significantly reduced shear bond strength: 10.87 → 3.78 MPa (0.5 mm) and 14.71 → 4.99 MPa (1 mm), both p = 0.00. Thickness increased SBS but not significantly (p = 0.110). Higher ARI scores (lower enamel-damage risk) in laser groups
4	Buu et al. (2010) [19]	IPS Empress Esthetic porcelain veneers; tested thickness approximately 0.72–1.50 mm	RelyX Veneer Cement A1 (3 M ESPE)	11 extracted anterior incisors in the main debonding trial (plus 6 veneers used as a learning set)	Er:YAG (2940 nm)	Laboratory study. Veneers were irradiated at 133 mJ/pulse, 10 Hz, 100 µs, with the fiber tip 3–6 mm from the veneer. Air/water spray was used in the learning set and air spray in the main set	All 11 veneers in the main trial were removed without damage to the underlying tooth structure. Debonding occurred mainly at the cement–veneer interface; when specimens were kept dry and air spray was used, 75% of veneers were removed without fracture
5	Walinski et al. (2021) [20]	Leucite-reinforced glass–ceramic veneers	Variolink Esthetic LC (Ivoclar Vivadent)	22 recently extracted human maxillary central incisors	Er,Cr:YSGG	Aimed to optimize debonding speed and thermal safety	As veneer thickness increases, more time is needed for removal; however, increasing thickness does not necessarily increase pulpal temperature. Calibration of power settings maximised efficiency
6	Al-Karadaghi & Jawad (2023) [21]	Lithium disilicate laminate veneers (IPS e.max Press HT), 0.7 mm thick	Light-cured resin cement (Variolink Esthetic LC, Ivoclar Vivadent)	30 extracted bovine mandibular incisors	Er,Cr:YSGG (fractional handpiece)	In vitro study. Six groups: five laser-irradiated (3–5 W in 0.5 W steps) plus control; 50 s exposure, 15 Hz; intrapulpal temperature, shear bond strength, ARI and SEM assessed	Laser significantly lowered shear bond strength at all power levels, even 3 W. Highest pulp temperature rise was 1.7 °C (5 W) — safe. No veneer fractures; favorable ARI scores (2–3)
7	Eid et al. (2021) [22]	Lithium disilicate and leucite-based glass ceramic veneers, 0.3 mm and 0.7 mm thick	Light-cured resin cement (MOJO Veneer Cement, Pentron)	84 extracted sound premolars	Er:YAG vs Er,Cr:YSGG (head-to-head comparison)	In vitro study. Three groups (Er:YAG/Er,Cr:YSGG/control), subdivided by ceramic type and thickness; debonding time recorded, surviving specimens tested for shear bond strength	Er,Cr:YSGG generally took longer to debond than Er:YAG. Lithium disilicate debonded faster than leucite-based ceramic with both lasers. Surviving specimens showed drastic SBS decrease vs. control
8	Giraldo-Cifuentes et al. (2020) [23]	Feldspathic porcelain veneer discs (IPS Empress Esthetic), 6 mm diameter × 0.8 mm thick	Light-cured resin cement (Variolink N LC, Ivoclar Vivadent)	75 extracted bovine teeth	Er,Cr:YSGG	In vitro study. Three groups (*n =* 25): 4 J/cm^2^ fluence, 2.7 J/cm^2^ fluence, and control (no laser); laser applied perpendicular at 4 mm distance for 60 s	Laser groups required far less force to debond than control (8.19 MPa control vs 0.91 MPa at 4 J/cm^2^ vs 0.48 MPa at 2.7 J/cm^2^, *p <* 0.001). Higher fluence needed more force than lower fluence. Adhesive failure rate increased with laser use (up to 96%)
9	Alikhasi et al. (2019) [24]	Feldspathic and lithium disilicate veneers	Variolink N LC (Ivoclar Vivadent)	57 bovine incisors	Er,Cr:YSGG	Investigated debonding time and dental pulp temperature	Variations in debonding time and pulp temperature based on veneer material and laser type. Minimal temperature rise during removal
10	El-Damanhoury et al. (2022) [25]	Lithium disilicate laminate veneers	Variolink Esthetic LC (Ivoclar Vivadent)	48 maxillary central incisors	Er:YAG	Investigated effect of laser power settings and veneer thickness on debonding time and pulpal temperature	Faster removal of thinner veneers; 5.4 W efficient for thicker veneers with minimal temperature changes. Higher power shortened debonding time but increased pulpal temperature
11	Iseri et al. (2014) [26]	Laminate veneers IPS Empress II	Variolink II	60 bovine mandibular incisor teeth	Er:YAG	Explored effect of Er:YAG laser on bonding strength of laminate veneers	Laser-assisted debonding maintained enamel integrity compared to mechanical methods. Results indicated impact of Er:YAG on bonding strength and clinical applications
12	Yilmaz et al. (2019) [27]	Lithium disilicate (IPS Empress II) discs, 0.5/1/2 mm thick	Panavia F (self-etch protocol) and RelyX ARC (total-etch protocol); Clearfil SE Bond used with the self-etch system	120 extracted human maxillary central incisors	Er,Cr:YSGG	In vitro study. Twelve groups by disc thickness, cementing agent, and laser use; laser applied at 5.5 W, 140 µs pulse duration, 20 Hz, for 180 s; shear bond strength and ARI evaluated	Debonding strength decreased with laser use and with decreasing disc thickness, reaching zero at 0.5 mm (complete dislodging, no extra force needed). Without laser, total-etch cement gave higher debonding values than self-etch
13	AlBalkhi, Swed & Hamadah (2018) [28]	Porcelain laminate veneers (lithium disilicate, IPS e.max), 0.7 mm thick	Light-cured resin cement (Variolink N, Ivoclar Vivadent)	40 extracted non-carious human maxillary premolars (16 used in main comparison)	Er:YAG (2940 nm)	In vitro study. Compared contact (CM) vs noncontact (NCM) application modes (360 mJ, 15 Hz), then tested additional NCM parameter groups at varying energy/frequency; debonding time and pulp temperature recorded	NCM was far faster than CM (12.6 s vs 96.4 s) but produced a higher temperature rise (4.2 °C vs 2.9 °C), both within safe limits. All veneers debonded successfully; failure modes were type 1 or 3 only
14	Amin (2023) [29]	Feldspathic porcelain (VITABLOCS Mark II), lithium disilicate CAD-CAM (IPS e.max CAD), and lithium disilicate press ceramic (IPS e.max Press), 0.5 mm thick, 5 mm diameter	Light-cured resin cement (RelyX Veneer A1, 3 M ESPE)	45 extracted human maxillary first premolars	Er,Cr:YSGG	In vitro study. Three groups by ceramic material; laser at 4.5 W, 60 µs pulse, 20 Hz, noncontact, 2 mm distance; debonding time measured, SEM failure analysis performed	Feldspathic porcelain took significantly longer to debond (10.07 s) than lithium disilicate CAD-CAM (5.13 s) or press (5.20 s), *p <* 0.001. No significant difference between the two lithium disilicate types. Failures mainly adhesive at the veneer–cement interface; no enamel damage
15	Zhang et al. (2018) [30]	Porcelain laminate veneers	RelyX Veneer 3 M Espe	12 freshly extracted teeth	Er:YAG	Ex vivo study on debonding of porcelain laminate veneers using Er:YAG laser	Provided insights into effectiveness of Er:YAG laser for debonding porcelain laminate veneers and influence on temperature increase
16	Giraldo Cifuentes et al. (2020) [31]	Lithium disilicate veneers	Variolink Esthetic N LC light-curing cement	68 bovine teeth	Er,Cr:YSGG	Examined effect on debonding of lithium disilicate veneers with four different thicknesses	Thinner veneers required less laser energy. Highlighted influence of laser type on debonding, particularly regarding veneer thickness
17	El-Sheikh et al. (2024) [32]	Ultrathin occlusal veneers, 0.5 mm: lithium disilicate (IPS e.max CAD), highly condensed lithium disilicate (GC Initial LiSi), and translucent zirconia (Katana STML)	Dual-cure resin cement (Breeze, Pentron)	24 extracted human upper first molars	Er,Cr:YSGG	In vitro study. Three groups by ceramic material; laser at 6 W, 20 Hz, scanning technique, 15 s; debonding time and post-debonding damage assessed	No significant difference in debonding time between the three materials. Lithium disilicate (e.max) and zirconia showed no damage; 40% of the highly condensed lithium disilicate (LiSi) samples fractured and 20% cracked
18	Al-Karadaghi et al. (2023) [33]	Lithium disilicate laminate veneers	Light-cured resin cement (Variolink N, Ivoclar Vivadent)	36 extracted non-carious human maxillary premolars	Er,Cr:YSGG	In vitro study. Explored influence of pulse duration and exposure time on debonding efficiency	Both laser modes safe for laminate veneer removal. Exposure time of 50 s and pulse duration of 60 µs yielded superior results
19	Raafat et al. (2025) [34]	Lithium disilicate (IPS e.max CAD) veneer specimens, 4 × 4 × 0.5 mm	Light-cured resin cement (Variolink Esthetic LC, Ivoclar Vivadent) with Scotchbond Universal adhesive	63 extracted human maxillary central incisors	Er,Cr:YSGG	In vitro study. Nine groups (*n =* 7) by power output (4/5/6 W) × water percentage (1/20/40%), plus unbonded control; intrapulpal temperature, debonding time, translucency and SEM evaluated	Highest temperature rise at 4 W/1% water (4.0 °C); lowest at 6 W/20% water (1.2 °C) — all within safe limits. Longest debonding time at 4 W/1% water (333 s); shortest at 6 W/20% water (17 s). 5 W/20% water recommended as an efficient, safe protocol if veneer reuse is intended; high power (6 W) reduced translucency
20	Zanini et al. (2021) [35]	Lithium disilicate (IPS e.max CAD) laminates, 3 × 3 × 0.7 mm, on enamel slabs	Three resin cements compared: Variolink Veneer, RelyX U200, RelyX Veneer	44 enamel slabs from 11 human third molars	Er,Cr:YSGG (two protocols: 3.5 W and 3.0 W, 20 Hz, noncontact)	In vitro study. Nine groups by cement × laser protocol (including non-laser controls); enamel evaluated via SEM, EDS and OCT before cementation and after debonding	No harmful morphological changes to enamel from laser irradiation; enamel prism changes seen only with total-etch cements (acid-conditioning effect, not laser heat). Both laser protocols were effective and safe; veneers debonded within 30 s
21	Yıldız et al. (2023) [36]	Lithium disilicate (IPS e.max CAD) slabs, 6 × 6 × 1 mm	RelyX Veneer (light-cured), Panavia V5 (dual-cured), and Multilink Automix (self-cured)	90 extracted bovine mandibular incisors	Er,Cr:YSGG	In vitro study. Three cement groups × three ageing conditions (no cycling, 5,000, 30,000 thermal cycles); laser at 3.5 W/10 Hz until debonding; debonding time, Vickers microhardness, SEM/EDS assessed	Self-cured cement took significantly longer to debond (22.7 s) than light-cured (10.8 s) or dual-cured (12.0 s). Ageing significantly reduced debonding time (no significant difference between 5,000 and 30,000 cycles). No microcracks or fractures observed
22	Abdulwahhab et al. (2025) [37]	Leucite-reinforced glass ceramic veneer discs (IPS Empress), 0.5 mm and 1 mm thick, 5 mm diameter	Light-cured resin cement (Variolink Esthetic LC, Ivoclar Vivadent) with Adhese Universal adhesive	40 extracted bovine mandibular incisors	Er,Cr:YSGG	In vitro study. Four groups (control/laser × two thicknesses, *n =* 10); laser at 2.5 W, 25 Hz, 60 s, scanning technique; shear bond strength, intrapulpal temperature and ARI evaluated	Laser significantly reduced shear bond strength at both thicknesses (9.94 → 4.66 MPa at 0.5 mm; 10.62 → 4.97 MPa at 1 mm, both *p <* 0.001). Higher ARI scores (less enamel-damage risk) in laser groups. Maximum pulp temperature rise was 2.6 °C — within the safety threshold
23	Çulhaoğlu et al. (2021) [38]	Feldspar ceramic (Vita Cerec Blocs), lithium disilicate (IPS e.max CAD), and resin nanoceramic (Lava Ultimate) laminate specimens, 3 × 3 mm, 0.5 mm and 1 mm thick	Resin cement (RelyX Veneer, 3 M ESPE)	120 extracted human maxillary central/lateral teeth	Er:YAG (2940 nm)	In vitro study. After thermocycling (10,000 cycles, 5–55 °C), each material/thickness group (*n =* 10) split into laser-treated vs control; laser at 150 mJ, 10 Hz, 100 µs, 1.5 W, 9 s, scanning; shear bond strength, SEM and ARI evaluated	Laser significantly weakened shear bond strength for all groups (*p <* 0.05); most dramatic decrease for IPS e.max at both thicknesses. 1 mm laser-treated specimens had higher SBS than 0.5 mm. Laser was less effective for the resin nanoceramic (Lava Ultimate), especially at 1 mm; no significant enamel damage observed
24	Xiong et al. (2025) [39]	Glass ceramic restorations (IPS e.max), 1 mm thick, 10 mm diameter, bonded to dentin	High-strength universal resin cement (Multilink N, Ivoclar Vivadent)	10 extracted third molars (dentin surfaces, occlusal enamel removed)	Er,Cr:YSGG (2794 nm): nanosecond-pulse vs microsecond-pulse modes compared	In vitro study. Two groups by pulse mode (3 mJ, 100 Hz; 100 ns vs 150 µs) for debonding the restoration and residual adhesive on dentin; pulp cavity temperature and dentin surface morphology (SEM) evaluated	Both pulse modes effectively debonded restorations. Nanosecond pulses caused a lower temperature rise (1.8 °C restoration/2.8 °C adhesive removal) than microsecond pulses (3.4 °C/5.8 °C, which exceeded the 5.5 °C safety threshold during adhesive removal). Nanosecond pulses produced more complete dentinal tubule opening, favorable for future bonding
25	Tak et al. (2015) [40]	N/A — resin cement discs irradiated through a lithium disilicate ceramic disc (IPS e.max CAD, 5 mm diameter, 1 mm thick), not a cemented tooth-veneer assembly	Five resin cements compared: G-Cem LinkAce, Multilink Automix, Variolink II, Panavia F 2.0, RelyX Unicem U100	None used — isolated cement/ceramic discs, no dental substrate	Er:YAG (600 mJ, 2 Hz/1.2 W, 45.4 J/cm^2^, through a 1 mm ceramic disc)	In vitro study. Ceramic disc placed between resin cement discs and the laser tip; cement discs irradiated with 2 pulses; volume loss measured via micro-CT before/after irradiation	All 5 cements were ablated by transmitted laser energy, but volume loss varied significantly: Multilink Automix (1.3 ± 0.1 mm^3^) and G-Cem LinkAce (1.1 ± 0.6 mm^3^) showed significantly higher ablation than RelyX Unicem (0.3 ± 0.07 mm^3^), Variolink II (0.4 ± 0.2 mm^3^), and Panavia F (0.6 ± 0.2 mm^3^), *p <* 0.05 — cement composition affects debonding susceptibility
26	Hussein et al. (2021) [41]	Lithium disilicate and feldspathic veneers	Two different curing mode resin cements	Bovine (number not specified)	Er,Cr:YSGG	Investigated debonding process with two different curing mode resin cements	No statistically significant difference in surface roughness (Ra) values between the two ceramics after debonding. Laser efficacy varied by veneer material and cement type
27	Van As (2012) [42]	Clinical removal of all-ceramic veneers and glass–ceramic restorations, including IPS Empress Esthetic and IPS e.max	Resin cement; exact cement varied/not consistently specified in the case report	Clinical case series; adult patients with anterior restorations in situ	Er:YAG	Clinical case report/series. Veneers were generally irradiated for about 30–60 s using Er:YAG; examples included 30 Hz, 175 mJ (5.25 W), 300 µs with air/water spray. Lithium disilicate crowns were also removed at 30 Hz, 200 mJ (6 W)	Laser-assisted removal enabled complete retrieval of ceramic restorations with limited risk to underlying tooth structure. Some restorations fractured, while others were removed intact; resin cement generally remained on the tooth
28	Kursoglu & Gursoy (2013) [43]	Fractured pressable ceramic laminate veneers (IPS Empress Esthetic) — 2 clinical cases	Resin luting cement (Variolink Veneer, Ivoclar Vivadent)	2 patients (clinical case report), anterior teeth in situ	Er:YAG	Clinical case report (not in vitro) of 2 patients with fractured laminate veneers removed via Er:YAG laser (20 Hz, 320 mJ, chisel-type tip, water irrigation, 9 s exposure)	Veneers removed without damage to underlying tooth structure in both cases. New restorations placed successfully, functioning satisfactorily at 3-year and 12-month follow-up
29	Deeb, Grzech-Lesniak & Bencharit (2023) [44]	Mixed ceramic restorations: lithium disilicate veneers and crowns, and zirconia crowns and fixed partial dentures	Various: bonded resin (Variolink Esthetics, Ivoclar), self-adhesive resin (RelyX Unicem 2, 3 M; Panavia SA Universal, Kuraray), zinc oxide eugenol; some cases unknown	29 patients; 46 prostheses/52 abutment units (44 natural teeth, 6 metal implant abutments, 2 zirconia implant abutments)	Er:YAG (*N =* 46) and Er,Cr:YSGG (*N =* 6)	Retrospective clinical analysis (not in vitro). Er:YAG at 170–420 mJ, 12–16 Hz, 2.5–5 W, QSP/SSP; Er,Cr:YSGG at 5 W, 15 PPS; laser applied noncontact, 5–8 mm distance, ~ 30 s per surface with air/water spray	50 of 52 abutments (> 95%) retrieved without damage. Retrieval time: veneers 2.25 ± 0.61 min, crowns 6.89 ± 8.07 min, FPDs 25 ± 10 min/abutment. Zirconia crowns took longer than lithium disilicate crowns (7.12 ± 8.91 vs 5.86 ± 2.41 min). 47.83% of prostheses recemented the same visit; no structural damage to ceramic or abutment observed

### Study characteristics

Al-Karadaghi et al. (2023) [33] found that both tested laser modes were safe for laminate veneer removal, with an exposure time of 50 s and a pulse duration of 60 µs yielding superior results. El-Damanhoury et al. (2022) [25] confirmed that higher laser power shortens debonding time but increases pulpal temperature, underscoring the need to balance efficiency with thermal safety; 5.4 W proved effective for thicker veneers with minimal temperature change.

AlBalkhi and Hamadah (2022) [16] showed that optimal energy density settings, combined with appropriate water/air cooling ratios, achieved efficient veneer removal with minimal heat generation. Walinski et al. (2021) [20] similarly found that increasing veneer thickness prolongs debonding time but does not necessarily raise pulpal temperature, provided power settings are carefully calibrated. Alikhasi et al. (2019) [24] and Giraldo Cifuentes et al. (2020) [31] both reported minimal temperature rise during Er,Cr: YSGG-assisted removal of ceramic veneers.

Giraldo Cifuentes et al. (2020) [31] established that thinner lithium disilicate veneers require less laser energy, improving precision and safety. Alikhasi et al. (2019) [24] observed variation in debonding time and pulpal temperature depending on veneer material (feldspathic vs. lithium disilicate). Hussein et al. (2021) [41] found no statistically significant difference in surface roughness between lithium disilicate and feldspathic veneers after Er,Cr:YSGG debonding, though efficacy varied by cement type.

AlBalkhi and Hamadah (2022) [16] demonstrated that the Er:YAG laser reduced residual bond strength in laminate veneers — a desirable outcome for efficient debonding. Iseri et al. (2014) [26] found that laser-assisted debonding maintained enamel integrity compared to mechanical methods, supporting its safety profile for clinical use. Zhang et al. (2018) [30] provided additional insights into Er:YAG effectiveness for porcelain laminate veneers and its influence on temperature during the procedure.

Oztoprak et al. (2012) [17] showed that short Er:YAG scanning periods significantly reduced the bond strength of ceramic laminate veneers, while Buu et al. (2010) [19] demonstrated that laser energy could pass through porcelain and act predominantly at the cement interface, allowing veneer removal without damage to the underlying tooth structure. Similar reductions in shear bond strength were reported by Al-Araji and Sulaiman (2022) [18] for lithium disilicate veneers of different thicknesses and by Al-Karadaghi and Jawad (2023) [21], who also maintained intrapulpal temperature within safe limits. Eid et al. (2021) [22] directly compared Er:YAG and Er,Cr:YSGG and found both effective, although removal time varied according to laser type, ceramic composition and thickness. In feldspathic veneers, Giraldo-Cifuentes et al. (2020) [23] found a marked reduction in debonding resistance after Er,Cr:YSGG irradiation, with failure occurring predominantly at the adhesive interface. Yilmaz et al. (2019) [27] further demonstrated that both ceramic thickness and cementation protocol influenced debonding, with thinner veneers requiring less force after irradiation. AlBalkhi, Swed and Hamadah (2018) [28] found non-contact Er:YAG application considerably faster than contact irradiation while maintaining pulpal temperature within safe limits. Amin (2023) [29] confirmed a strong material-dependent effect, as feldspathic porcelain required longer removal times than either pressed or CAD-CAM lithium disilicate. van As (2012) [42] and Kursoglu and Gursoy (2013) [43] extended these observations to clinical situations, demonstrating that erbium lasers could assist removal of ceramic veneers while preserving the underlying tooth tissues. El-Sheikh et al. (2024) [32] reported comparable debonding times among ultrathin lithium disilicate, LiSi and zirconia occlusal veneers, although LiSi restorations showed a higher incidence of cracks and fractures after removal.

Raafat et al. (2025) [34] showed that laser power and water cooling jointly influenced both debonding time and pulpal temperature, emphasizing the need to balance efficiency with thermal safety. Zanini et al. (2021) [35] found no relevant laser-induced enamel damage, whereas Yıldız et al. (2023) [36] demonstrated that resin cement polymerization mode and ageing significantly affected removal time. Abdulwahhab et al. (2025) [37] similarly reported reduced shear bond strength of leucite-reinforced veneers without exceeding the thermal safety threshold. Çulhaoğlu et al. (2021) [38] found that Er:YAG effectiveness varied according to restorative material and thickness, while Xiong et al. (2025) [39] showed that nanosecond Er,Cr:YSGG pulses produced lower temperature increases than microsecond pulses during ceramic restoration and adhesive removal. Tak et al. (2015) [40] demonstrated that resin cement composition influenced susceptibility to laser ablation. Finally, Deeb, Grzech-Lesniak and Bencharit (2023) [44] provided retrospective clinical evidence, reporting successful retrieval of more than 95% of ceramic restorations and showing that removal time was affected by restoration type, ceramic material and cement characteristics.

### Quantitative parameter synthesis across studies

To allow direct comparison across the evidence base, quantitative laser parameters and outcomes are reported exactly as available in the primary studies. Fixed irradiation protocols used only for bond-strength testing are not presented as mean debonding times (Table 6).

**Table 6:** Quantitative parameter synthesis across studies

Study	Laser Cohort	Power Settings (W)	Pulse Duration/Cooling	Debonding/irradiation time (s), as reported	Pulpal temperature increase (°C), as reported
AlBalkhi & Hamadah (2022) [16]	Er:YAG	4.05 W (270 mJ × 15 Hz; derived)	50, 100, 300 µs/water:air 1:1 or 3:3	All veneers debonded. Group means ranged from 7.4 to 104.6 s; 50/100 µs groups: 7.4–17.0 s; 300 µs group up to 104.6 ± 11.8 s	Group mean ΔT values ranged 0.6–3.4 °C; highest 3.4 ± 0.6 °C
Oztoprak et al. (2012) [17]	Er:YAG	5.0 W (100 mJ × 50 Hz)	Pulse duration NR/cooling NR	NM as spontaneous time-to-debond. Fixed irradiation durations: 3, 6 and 9 s; SBS was the endpoint	NM
Al-Araji & Sulaiman (2022) [18]	Er,Cr:YSGG	2.5 W (100 mJ × 25 Hz)	60 µs/30% water, 70% air	NM as spontaneous time-to-debond. Fixed irradiation: 60 s; SBS/ARI endpoints	NM
Buu et al. (2010) [19]	Er:YAG	1.33 W (133 mJ × 10 Hz; derived)	100 µs/Set A air–water; Set B air only	Main set (Set B): 68 ± 79 s [3–290]. Learning set (Set A): 51 ± 68 s. In Set B, intact veneers: 138 ± 82 s [51–290]; fractured veneers: 21 ± 27 s to first fracture [3–60]	NM
Walinski et al. (2021) [20]	Er,Cr:YSGG	10.0 W (333 mJ × 30 Hz)	Pulse duration NR/80% air, 50% water	Abstract: 14.16 ± 0.60 s [10.75–21.25]. Results text reports 14.71 ± 3.05 s [11.5–21.25]	Abstract: 0.71 ± 0.15 °C. Results text reports 0.85 ± 0.88 °C
Al-Karadaghi & Jawad (2023) [21]	Er,Cr:YSGG	3.0–5.0 W	Pulse duration/cooling ratio NR in accessible report; fractional technique	NM as spontaneous time-to-debond. Fixed irradiation: 50 s for each laser-treated group; SBS was the endpoint	Highest reported ΔT: 1.7 °C at 5 W; pooled mean NR
Eid et al. (2021) [22]	Er:YAG and Er,Cr:YSGG	6.0 W for both (300 mJ × 20 Hz)	Er:YAG: 100 µs; Er,Cr:YSGG: 60 µs/both 80% water, 60% air	Median [range], s. Er,Cr:YSGG: LD 0.3 mm 6.3 [5JABB7.3], LD 0.7 mm 7 [6–8], leucite 0.3 mm 9.5 [9JABB11], leucite 0.7 mm 13 [12.6–14]. Er:YAG: 3.5 [2.5–5], 5.6 [3–7], 8.25 [7JABB10], 12 [9JABB14], respectively	NM
Giraldo-Cifuentes et al. (2020) [23]	Er,Cr:YSGG	3.0 W (50 Hz) and 4.0 W (100 Hz)	140 µs/20% water, 10% air	NM as spontaneous time-to-debond. Fixed irradiation: 60 s; SBS/failure mode endpoints	NM
Alikhasi et al. (2019) [24]	Er,Cr:YSGG	2.5 W at 25 Hz	60 µs/cooling ratio NR	Mean ± SD [range], s: feldspathic 103.68 ± 26.76 [60–170]; lithium disilicate MO 106.58 ± 47.22 [10–213]; lithium disilicate HT 103.84 ± 32.90 [47–178]	ΔT < 1 °C in all groups; exact group means NR
El-Damanhoury et al. (2022) [25]	Er:YAG	1.5, 3.0 and 5.4 W at 10 Hz	100 µs/water irrigation 40 mL/min; air/aspiration 40 mL/min	Mean ± SD, s (0.5 mm/1.0 mm): 1.5 W: 27.58 ± 7.97/199.12 ± 44.45; 3.0 W: 4.72 ± 2.43/14.05 ± 5.00; 5.4 W: 2.10 ± 0.86/5.10 ± 2.73	Mean ΔT, °C (0.5/1.0 mm): 1.5 W: 0.7 ± 0.20/5.7 ± 1.79; 3.0 W: 0.4 ± 0.10/2.1 ± 0.75; 5.4 W: 0.4 ± 0.23/0.8 ± 0.48
Iseri et al. (2014) [26]	Er:YAG	5.0 W (100 mJ × 50 Hz)	Pulse duration NR/no water	NM as spontaneous time-to-debond. Fixed irradiation: 9 s; SBS was the endpoint	NM
Yilmaz et al. (2019) [27]	Er,Cr:YSGG	5.5 W (275 mJ × 20 Hz)	140 µs/80% water, 90% air	NM as spontaneous time-to-debond. Fixed irradiation: 180 s; SBS/ARI endpoints	NM
AlBalkhi et al. (2018)[28]	Er:YAG	5.4 W main CM/NCM comparison (360 mJ × 15 Hz); additional NCM groups 3.0–4.05 W	100 µs/water:air 1:1	Main comparison: non-contact 12.6 s vs contact 96.3 s	Main comparison: non-contact 4.2 °C vs contact 2.9 °C
Amin (2023) [29]	Er,Cr:YSGG	4.5 W at 20 Hz	60 µs/80% water, 60% air	Mean ± SD [range], s: feldspathic 10.067 ± 1.668 [8JABB12]; lithium disilicate press 5.200 ± 1.146 [4JABB7]; lithium disilicate CAD-CAM 5.133 ± 1.125 [4JABB7]	NM
Zhang et al. (2018) [30]	Er:YAG	3.0 W (100 mJ × 30 Hz)	800 µs/abundant air–water spray, 4/4 ratio	Paper reports pulses rather than seconds: 9836.25 ± 4501.91 pulses. Derived at 30 Hz: 327.9 ± 150.1 s [135.9–571.9]	NM
Giraldo Cifuentes et al. (2020) [31]	Er,Cr:YSGG	4.0 W at 50 Hz	140 µs/20% water, 20% air	NM as spontaneous time-to-debond. Fixed irradiation: 60 s; SBS was the endpoint	NM
El-Sheikh et al. (2024) [32]	Er,Cr:YSGG	6.0 W at 20 Hz	Pulse duration NR/80% water, 60% air	Mean ± SD [range], s: IPS e.max CAD 86.94 ± 20.63 [58.80–109.30]; GC Initial LiSi 86.14 ± 25.16 [55.00–123.70]; Katana STML 87.52 ± 20.45 [60.40–108.60]	NM
Al-Karadaghi et al. (2023) [33]	Er,Cr:YSGG	3.0 W	60 µs (H mode) or 700 µs (S mode)/60% air, 40% water	NM as spontaneous time-to-debond. Fixed exposure subgroups: 20, 30, 40, 50 and 60 s; SBS was measured after irradiation	Maximum reported ΔT: 2.8 °C with 60 µs vs 0.6 °C with 700 µs. At 60 µs, 50 s and 60 s exposures increased temperature by about 2.0 °C and 2.7 °C
Raafat et al. (2025) [34]	Er,Cr:YSGG	4.0, 5.0 and 6.0 W at 15 Hz	Pulse duration NR/20% air; water 1%, 20% or 40%	Mean ± SD, s. 4 W: 333.4 ± 74.8 (1%), 131.0 ± 14.6 (20%), 137.0 ± 21.1 (40%). 5 W: 62.8 ± 12.7, 60.4 ± 18.1, 130.4 ± 17.8. 6 W: 61.0 ± 13.6, 17.0 ± 5.9, 59.0 ± 14.1	Mean ΔT, °C. 4 W: 3.2 (1%), 3.4 (20%), 1.8 (40%). 5 W: 4.0, 1.6, 2.6. 6 W: 3.4, 1.2, 2.0. Lowest 1.20 ± 0.45 °C at 6 W/20%; highest 4.00 ± 0.00 °C at 5 W/1%
Zanini et al. (2021) [35]	Er,Cr:YSGG	3.5 W and 3.0 W, both at 20 Hz	Pulse duration/cooling NR; non-contact protocols	NR as a quantitative time outcome; study focused on enamel morphological, optical and elemental changes after laser debonding	NM (temperature not directly measured)
Yıldız et al. (2023) [36]	Er,Cr:YSGG	3.5 W at 10 Hz	Pulse duration NR/air only, no water	Mean ± SD by cement: light-cure 10.83 ± 7.28 s; dual-cure 12.0 ± 7.96 s; self-cure 22.67 ± 12.68 s. By ageing: no ageing 22.33 ± 13.62; 5000 cycles 11.83 ± 7.52; 30,000 cycles 11.83 ± 7.26. Overall observed range 3–65 s	NM
Abdulwahhab et al. (2025) [37]	Er,Cr:YSGG	2.5 W (100 mJ × 25 Hz)	60 µs/70% air, 30% water	NM as spontaneous time-to-debond. Fixed irradiation: 60 s; SBS/ARI endpoints	Mean ΔT: 1.36 °C for 0.5-mm veneers and 1.71 °C for 1.0-mm veneers. Maximum individual increases: 2.6 °C and 2.2 °C, respectively
Çulhaoğlu et al. (2021) [38]	Er:YAG	1.5 W (150 mJ × 10 Hz)	100 µs/60% water, 40% air	NM as spontaneous time-to-debond. Fixed irradiation: 9 s; SBS was the endpoint	NM
Xiong et al. (2025) [39]	Er,Cr:YSGG	0.3 W (3 mJ × 100 Hz; derived)	100 ns (= 0.1 µs) vs 150 µs/no water mist cooling	NR as a debonding-time outcome; both pulse modes achieved restoration debonding and subsequent residual-adhesive removal	Reported maximum/observed ΔT: restoration debonding 1.8 °C (100 ns) vs 3.4 °C (150 µs); residual adhesive removal 2.8 °C vs 5.8 °C
Tak et al. (2015) [40]	Er:YAG	1.2 W (600 mJ × 2 Hz)	1000 µs/cooling NR	NA: bench ablation model, not a veneer-tooth debonding model. Resin cement discs were irradiated through a ceramic disc with 2 pulses	NA
Hussein et al. (2021) [41]	Er,Cr:YSGG	4.5 W at 20 Hz	60 µs (H mode)/60% air, 80% water	NR/NM as a quantitative debonding-time outcome. Laser was applied by scanning until a change indicating debonding; study outcomes were mainly surface topography and roughness	NM
van As (2012) [42]	Er:YAG	5.25 W in veneer cases (175 mJ × 30 Hz); author generally used 5–6 W	300 µs in reported veneer cases/air–water spray	No study-wide mean. Case 1: 30–45 s irradiation. Author reports typical porcelain veneer/crown irradiation approximately 45–60 s before mechanical completion	NM
Kursoglu & Gursoy (2013) [43]	Er:YAG	6.4 W (320 mJ × 20 Hz; derived)	200 ms as printed in the article (unit internally inconsistent)/water irrigation	Clinical case report: fixed 9 s irradiation in the reported cases; not a group mean	NM
Deeb et al. (2023) [44]	Er:YAG and Er,Cr:YSGG	Er:YAG 2.5–5.0 W (170–420 mJ, 12–16 Hz); Er,Cr:YSGG 5.0 W at 15 PPS	Er:YAG: QSP/SSP, exact µs NR/air–water 2/2 or 6/6. Er,Cr:YSGG: pulse duration NR/20% air, 20% water	Clinical retrospective series. Veneers: 2.25 ± 0.61 mi*n =* 135 ± 36.6 s (converted). Crowns and FPDs required longer; no veneer range reported	NM

• *NR* not reported in the available primary report, *NM* not measured as an outcome, *NA* not applicable

• Where power in watts was calculated from pulse energy × frequency, the value is marked as derived

• For studies using a fixed irradiation duration followed by shear bond strength testing, the fixed exposure is reported but is not interpreted as a mean time-to-debond

• Zhang et al. (2018) [30]: time in seconds is derived from the reported pulse count at 30 Hz; the primary paper reports pulse counts

• Walinski et al. (2021) [20]: the abstract reports 14.16 ± 0.60 s and 0.71 ± 0.15°C, whereas the results text reports 14.71 ± 3.05 s and 0.85 ± 0.88°C; both are retained transparently

• Kursoglu & Gursoy (2013) [43]: the original article prints a pulse width of 200 ms. This value is retained as reported, although the unit is internally inconsistent with a 20-Hz repetition rate

• Deeb et al. (2023) [44]: veneer retrieval time was reported as 2.25 ± 0.61 min and converted here to 135 ± 36.6 s for comparability

## Discussion

### Summary of included clinical evidence

The studies support the integration of erbium lasers into veneer debonding workflows, with both Er:YAG and Er,Cr:YSGG systems demonstrating clinical viability. Sayed et al. (2022) [45] documented the effectiveness of Er,Cr:YSGG in removing various ceramic veneer materials without substantial damage to underlying tooth structure, with parameters adjustable to minimize thermal effects. Saremi et al. (2023) [46] further demonstrated that Er,Cr:YSGG lasers offer superior precision in controlled energy delivery, mitigating overheating risk and improving patient outcomes and also noted that different ceramic materials exhibit varying responses to laser debonding, requiring tailored settings for optimal results, consistent with Giraldo Cifuentes et al. (2020) [31].

Multiple studies highlight three interconnected advantages of laser-assisted debonding: precision, patient comfort, and efficiency. Er and Er,Cr lasers can selectively target the resin adhesive without damaging the underlying tooth structure or causing significant thermal effects [19, 25, 33, 35]. A consistent theme across the included studies is that laser efficacy is not universal — it depends on the specific veneer material, its thickness, and the resin cement employed.

### Thermal safety considerations

Preserving the vitality of the dental pulp is a critical parameter in debonding procedures. An increase of pulp temperature over the 5.6 °C limit is generally considered to induce irreversible thermal damage. The included studies prooved that increasing laser power accelerates veneer removal but causes higher intrapulpal temperature spikes [33, 34].

Thermal safety was found not to be determined by a single parameter, but also by other settings such as power, pulse duration, and active cooling. AlBalkhi and Hamadah [16] confirmed that the air/water cooling ratio is critical to prevent heat accumulation. Walinski et al. [20] further demonstrated that the removal of thicker veneers do not necessarily produced higher pulpal temperatures, when power output is carefully calibrated alongside active water irrigation.

### Influence of veneer thickness and ceramic material

The interaction between laser energy and the restoration is influenced by the restorative material’s thickness and optical properties. Thinner veneers require shorter laser action time and lower power to achieve restauration detachment. For instance, El-Damanhoury et al. [25] and Walinski et al. [20] both observed that the higher ceramic thickness influences the laser efficiency on the resin cement.

Also ceramic composition may impact energy transmission and absorption coefficients. Alikhasi et al. [24] mentioned different debonding rates between lithium disilicate and feldspathic glass–ceramics, whereas Hussein et al. [41] found that cement type altered overall laser efficacy, post-debonding surface topography did not differ significantly between these two ceramic types.

### Laser debonding efficiency

Comparing laser efficiency with mechanical removal erbium lasers provide a more rapid and less harmful debonding process than the rotary burs by reducing the risk of enamel damage.

This research showed that both Er:YAG and Er,Cr:YSGG laser systems are efficient in removing ceramic veneers by reducing the bond strength. Biophysical mechanisms like thermal ablation and photoablation cause rapid degradation of the resin cement layer without altering the ceramic substrate [23].

When comparing erbium lasers, althogh studies showed differences in procedural efficiency, both systems demonstrated successful veneer removal.They showed high water absorption and lower absorption in dental hard tissue,but while Er,Cr:YSGG lasers systems operate at a wavelength of 2780 nm, which aligns closely with the absorption bands of water and residual monomers in resin cements, Er:YAG systems operate at 2940 nm, matching the absolute peak of water absorption [22].

Regarding debonding time,studies showed that Er,Cr:YSGG lasers achieved rapid debonding times, without significant differences in different ceramic materials [24]. Thinner veneers required shorter irradiation times and lower energy delivery than thicker restorations [18, 25, 27].

The lack of standardisation in pulse duration, energy density, and beam movement patterns make difficult to define the superiority of one wavelength over the other. The adequate adjustment of parameter settings influences lasers efficiency more than the specific wavelength selected [16, 20].

### Clinical implications

Laser energy delivery may also improve patient comfort by minimizing tissue trauma and nerve stimulation, thereby reducing pain per ception and anxiety compared with mechanical methods [33]. In addition, optimized laser parameters can reduce debonding time and chairside duration, improving practice without compromising tooth structure and care quality [25, 33] Mechanical degradation facilitated veneer separation by structural degradation of the resin layer occurred via thermal ablation or photoablation without requiring direct, aggressive force against the underlying enamel substrate [23].

Although these laboratory results are encouraging into clinical practice should be approached cautiously because all included evidence originates from controlled experimental settings, so clinicians must use them as baseline references rather than definitive clinical protocols.

The significant upfront cost of laser systems remains a barrier, particularly for smaller practices where investment in CAD/CAM technologies may take precedence [1]. Adequate training is essential: without proper calibration and technique, lasers risk causing soft tissue damage, enamel loss, and thermal injury [47]. Dentists must stay current with evolving laser technologies and their interaction with specific materials to minimize iatrogenic risk.

### Limitations

The primary limitation of the current evidence base is that most of the included studies are laboratory-based (in vitro or ex vivo) lacking robust clinical trials that are important for clinical outcomes. In order to solve these inconsistence, future research must focus on clinical trials that assess clinical efficiency and post-operative outcomes in patients (comfort, treatment acceptance,pulp vitality and long-term safety). Standardized guidelines for laser parameters are necessary for energy density, pulse formatting, water flow rates, and specific handpiece distances to allow for reproducible protocols and objective evaluation. Well-designed clinical studies are necessary before definitive clinical recommendations can be established. Prospero registration was not completed prior to the start of data collection, which is a requirement for registration.

## Conclusions

This systematic review identified encouraging laboratory evidence supporting the use of Er:YAG and Er,Cr:YSGG lasers for ceramic veneer detachment. Debonding efficiency was influenced by veneer thickness, ceramic type, cement composition, and irradiation parameters,while thermal changes remained acceptable when appropriate settings were used.,

Laser systems offer significant advantages over traditional mechanical methods: greater precision, enhanced patient comfort, reduced procedural time, and preservation of tooth structure. These benefits position laser-assisted debonding as a valuable and increasingly justified component of modern restorative dental practice.

Continuous investment in research, training, and interdisciplinary collaboration will be essential to accelerate the adoption of these techniques and deliver safer, more predictable outcomes for patients.

## Data Availability

No datasets were generated or analysed during the current study.
